# Cochlear isolation in neonatal mice for *in vitro* and *ex vivo* models: an anatomical landmark-based protocol

**DOI:** 10.3389/fphar.2026.1727526

**Published:** 2026-05-20

**Authors:** Eugenue V. Polikarpov, Sergey V. Kozin, Elena Smolyarchuk, Kirill Savostyanov, Artem V. Mirontsev, Susanna Sologova, Veronika V. Chasovnikova, Ksenia V. Eremeeva, Andrey Fisenko, Dmitry Kudlay, Zanda Bakaeva

**Affiliations:** 1 Institute of Pharmacy, FSAEI HE I.M. Sechenov First MSMU of MOH of Russia (Sechenovskiy University), Moscow, Russia; 2 National Medical Research Center of Children’s Health, Russian Ministry of Health, Moscow, Russia; 3 Research and Clinical Center for Otorhinolaryngology FMBA of Russia, Moscow, Russia; 4 Lomonosov Moscow State University, Moscow, Russia; 5 National Research Center — Institute of Immunology FMBA of Russia, Moscow, Russia; 6 Kalmyk State University Named After B.B. Gorodovikov, Elista, Russia

**Keywords:** auditory neurons, cochlear explant, cochlear isolation, hair cell, inner ear, otic capsule, temporal bone

## Abstract

**Introduction:**

*Ex vivo* and *in vitro* models utilizing inner ear tissue from neonatal rodents are vital for developing treatments for hearing loss caused by damage to hair cells or auditory neurons. The cochlear isolation from the skull is associated with a high risk of mechanical damage to the soft tissues enclosed in the otic capsule. Such damage may result in artifactual hair cell loss and morphological alterations of SGNs. This stage of cochlear dissection requires more detailed description. To overcome technical difficulties, we described a protocol based on the use of key anatomic lines of the skull during dissection.

**Methods:**

We propose an optimized stepwise technique for cochlear isolation based on dissection of skull bones along specific anatomical skull lines—the spheno-occipital, spheno-petrosal, and petro-occipital synchondroses, along with the petrosquamosal suture. This approach utilizes targeted dissection along natural anatomical skull lines to carefully separate skull bones, reducing excess tissue and unnecessary manipulation of the cochlea. Sample preservation was assessed through immunofluorescence staining for hair cells (phalloidin) and spiral ganglion neurons (β-III tubulin) in both *ex vivo* and *in vitro* models.

**Results:**

The technique significantly reduced superfluous tissue adherence, thereby facilitating subsequent processing of the cochlear surface. Intact cochleae were consistently obtained with preserved auditory capsules and the structure of the membranous labyrinth. Immunofluorescence analysis confirmed the preservation of inner ear samples obtained for creating *in vitro* and *ex vivo* models. The protocol allows cochlear isolation in approximately 3 min, depending on the researcher’s skill, with a success rate of 92.9% (based on 70 dissections). Modiolus isolation takes approximately 7 min.

**Conclusion:**

Cochlear isolation is a technically challenging procedure that requires both practiced skill and careful execution. A key feature of our protocol lies in the emphasis on specific anatomical landmarks, which facilitates a clearer understanding of the cochlear isolation process and prevents actions that would compromise the integrity of the cochlea. This is important for researchers who are developing the skills necessary to create *in vitro* or *ex vivo* cochlear models. The described technique may potentially accelerate cochlear isolation, although its effectiveness largely depends on the researcher’s skill and experience.

## 1 Introduction

More than 5% of the world’s population suffers from hearing impairment, which amounts to about 466 million people (Chadha et al., 2021). Sensorineural hearing loss is a common form of hearing impairment (Sheffield and Smith, 2019). This pathology may result from various factors, primarily including excessive noise exposure, age-related changes, ototoxic drug use, infections and genetic disorders (Tripathi and Deshmukh, 2022). Sensorineural hearing loss is characterized by irreversible loss and/or dysfunction of hair cells, spiral ganglion neurons, and degeneration of cochlear ribbon synapses (Liberman and Kujawa, 2017). The pathogenesis may also involve supporting cells and stria vascularis cells (Wan et al., 2013; Yu et al., 2021).

It should be emphasized that mechanosensory hair cells in the mammalian cochlea form only during embryonic development, and their regeneration in mature individuals is impossible. Spiral ganglion neurons also lack regenerative capacity in adult mammals. The potential for regeneration of these structures is currently under active investigation (Zhang et al., 2024; Qi et al., 2024).

Ototoxic drugs occupy a special place among the causes of sensorineural hearing loss. Aminoglycoside antibiotics and platinum-based chemotherapeutic agents, such as cisplatin and carboplatin, exhibit the most potent ototoxic effects. Other drugs that are known to demonstrate ototoxicity include loop diuretics, macrolides, antimalarials and non-steroidal anti-inflammatory drugs (NSAIDs) (Reynard and Thai-Van, 2024).

To date, sodium thiosulfate remains the only clinically approved otoprotective agent worldwide, specifically indicated for preventing cisplatin-induced ototoxicity (Freyer et al., 2017). In this regard, the development of new otoprotective strategies that do not reduce the antitumor activity of drugs remains relevant (Tan et al., 2020).

Various experimental models are used to develop genetic and pharmacological approaches for preventing and treating sensorineural hearing loss. The most commonly used *in vivo* models are mice, rats, guinea pigs, chinchillas, and zebrafish. In hearing research, *ex vivo* models are widely used, including whole cochlear, organ of Corti and spiral ganglion explants (Buswinka et al., 2023; Sun et al., 2021). *In vitro* models frequently utilize cochlear cell lines (e.g., House Ear Institute-Organ of Corti 1 (HEI-OC1) cells), isolated spiral ganglion cells and auditory sensory epithelial cells (Yorgason et al., 2011; Wu et al., 2023).

Cochlear isolation and inner ear tissue processing can be performed in experimental animals of varying ages (Meas et al., 2018). *In vitro* and *ex vivo* modeling, cochleae from neonatal mice or rats are most commonly used, as the skull bones and cochlea have not ossified at this age, making the membranous labyrinth structures relatively easy to isolate from the otic capsule (Hahnewald et al., 2016). Microdissection of the cochlea of an adult animal presents significant technical challenges. After P14, complete ossification of the murine labyrinthine capsule makes its removal difficult while preserving the integrity of the soft tissue structures of the cochlea (Meas et al., 2018; Landegger et al., 2017; Fang et al., 2019). In this regard, establishing cochlear systems *in vitro* and *ex vivo* from adult samples requires further optimization (Li et al., 2022).

Cochlear dissection can be conceptually divided into two main steps: isolation of the cochlea from the temporal bone followed by processing of the inner ear tissue. A critical parameter is the interval between animal decapitation and either the incubation of cochlear samples or their placement in the experimental medium (Parker et al., 2010). These procedures require substantial practical experience and a refined technique in order to minimize the risk of damaging the samples.

Studies detailing cochlear dissection protocols have predominantly focused on processing of the inner ear tissue. This phase involves direct instrument contact with membranous labyrinth structures and carries the highest risk of damaging them. It should be emphasized that the quality and integrity of cochlear samples for *in vitro* or *ex vivo* modeling also depend on the step of isolating the cochlea from surrounding cranial tissues. Dissection of surrounding tissues carries a risk of mechanical damage to the otic capsule. Any deformation of the cartilaginous wall of the otic capsule may lead to loss of integrity of the membranous labyrinth structures. To prevent damage to the otic capsule and the membranous labyrinth, adjacent anatomical structures must be carefully controlled. To reduce the risk of mechanical damage to the cochlea, we developed an optimized dissection protocol.

More than 50 years ago, Sobkowicz et al. (1975) developed a method for culturing the organ of Corti (Sobkowicz et al., 1975), later complemented by more detailed dissection techniques (Sobkowicz et al., 1993). Those studies used anatomical landmarks such as the anterior and posterior cranial fossae, but mostly to locate the cochlea rather than as guides for dissection. Our study instead uses synchondroses and sutures as anatomical guides for cochlear isolation. This helps reduce mechanical stress on the otic capsule and preserves the integrity of the membranous labyrinth. This work fills a methodological gap by providing a clear anatomical rationale for each step of the isolation procedure. In doing so, it complements existing protocols and makes *ex vivo* and *in vitro* models more accessible for pharmacological and genetic studies of hearing loss.

The aim of this study is to optimize the protocol for isolating the cochlea from the temporal bone of neonatal mice for subsequent cochlea surface preparation. In this paper, we propose a technique for cochlear isolation in neonatal mice and believe that this approach may be particularly useful for those who are mastering the technique of cochlear isolation and processing of the inner ear tissue.

## 2 Materials and methods

We analyzed existing protocols for isolating and processing of the mammalian cochleae tissue, focusing specifically on methodological articles with video demonstrations that describe the isolation and processing of the neonatal mammalian cochlea (Table 1). Our goal was to identify critical steps in the cochlear isolation procedure that require both optimization and more detailed description. The literature search was performed using open-access sources available in electronic databases and search engines: PubMed, Scopus, Web of Science and Google Scholar.

**TABLE 1:** Key components of cochlear isolation techniques in different studies.

Source	Animal model (age)	Dissecting solution	Dissecting tools
Parker et al. (2010)	Mice (P4)	HBSS	#11 Scalpel Blade (BD Biosciences, Cat# 372611); Dumont #4 Forceps (Fine Science Tools, Germany, Cat# 11241-30); Dumont #55 Forceps (Fine Science Tools, Germany, Cat# 11295-51); Operating scissors (Roboz Surgical Instruments Cat# RS-6806)
Haqu et al. (2015)	Mice (E13)	HBSS	Minutien pins (Fine Science Tools, Germany, Cat# 26002-15) Dumont #5 forceps (Fine Science Tools, Germany, Cat# 11251-10)
Landegger et al. (2017)	Mice (P3-P5)	HBSS	Operating scissors (Roboz Sur-gical Instruments Cat# RS-6806); Scalpel Blades - #15 (Fine Science Tools, Germany, Cat# 10015-00); Scalpel Handle - #4 Fine Science Tools, Germany, Cat# 10004-13); Dumont #4 Forceps (Fine Sci-ence Tools, Germany, Cat# 11241-30)
Levano et al. (2023)	Mice, Rat (P3-P5)	PBS	Operating scissors (Fine Science Tools, Germany, Cat# 14005-16); Operating scissors (Fine Science Tools, Germany, Cat# 14088-10); Operating tweezers (Fine Science Tools, Germany, Cat# 11008-15); Scalpel Handle #4 (Fine Science Tools, Germany, Cat# 10004-13); Surgical Scalpel Blade no. 23 (Swann Morton, England, Cat# 210)

Animal experiments were performed in accordance with the European Convention for the Protection of Vertebrate Animals used for Experimental and other Scientific Purposes and with the Good Laboratory Practice Rules approved by Order No. 199n of the Ministry of Health of the Russian Federation (4 January 2016). The experiments used mice on postnatal days 1–2 (P1–P2). All procedures were performed under sterile conditions.

### 2.1 Stepwise procedure

The protocol is based on the use of key anatomical lines of the skull base: the spheno-occipital synchondroses (SOs), sphenopetrosal sutures (SPs), petro-occipital synchondroses (POs), and the petrosquamosal (PS) fissure. In neonatal mice, the synchondroses are still non-ossified and can be used as dissection lines. Dissection along these natural boundaries allows access to the cochlea while minimizing mechanical impact on the otic capsule and reducing excess surrounding tissue. This increases the likelihood of preserving the integrity of the membranous labyrinth structures.

#### 2.1.1 Preparation of the work area and solutions for dissection

Disinfect the work surface with 70% ethanol. Prepare a set of dissection instruments, including ultra-fine forceps Dumont #4 (Fine Science Tools, Cat# 11241-30), Dumont #55 (Fine Science Tools, Cat# 11295-51) and a microdissection knife (Fine Science Tools, Cat# 10056-12), dissecting dishes (Greiner Bio-One, Cat# 627160), a cooling agent (e.g., ice or refrigerant) and a sealed plastic bag or container for disposing of the carcass.

For the cochlear isolation procedure, use a sterile, chilled balanced salt solution (e.g., Hanks’ balanced salt solution (HBSS) without calcium and magnesium (Gibco, Thermo Fisher Scientific, Cat# 14185-052), or PBS (Sigma-Aldrich, Cat# P4417-100TAB). Pour 3–4 mL of the solution into a dissecting dish and place it on a cooling platform. Keep the dissecting dishes on the cooling platform throughout the entire sample preparation process. The dissection station should be equipped with a stereomicroscope (Stereomicroscope Olympus SZX7, Olympus Corporation) and an illuminator (Fiber-Lite Illuminator, RWD Life Science, Cat# 76301). Depending on availability and personal preference, researchers may use alternative instruments.

#### 2.1.2 Localization of otic capsules in the skull base

**FIGURE 1:**
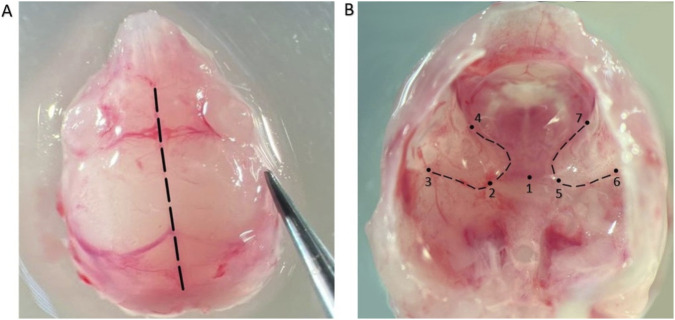
Images were obtained using a stereoscopic microscope. **(A)** Skull opening line: the dashed line indicates the skull opening trajectory along the sagittal suture (magnification 8X). **(B)** Skull base: arrows indicate the localization of otic capsules, the dashed line indicates the cochlear part of the otic capsule (magnification 16X). Dissecting movements of the forceps are performed from the initial forceps position (1) along the lines 2–5 (SOs), 2–3 and 5–6 (SPs), and 2–4 and 5–7 (POs).

Decapitate the neonatal mouse using sterile dissecting scissors and place the head in a dissecting dish containing HBSS or PBS. Remove the skin from the skull using a surgical blade or spring scissors. The skull bones are not yet ossified at this stage of development. This makes them easy to dissect and facilitates isolation of the cochlea. Open the cranial cavity along the sagittal line with a scalpel, then insert a spatula to gently separate brain tissue from the skull bones (Figure 1A). After removing the brain tissue, locate the otic capsule under a stereomicroscope (8–1×6 magnification) (Figure 1B). The otic capsule of neonatal mice is non-ossified and highly fragile. Its translucent cartilaginous structure allows the membranous labyrinth to be visualized. These characteristics make it clearly distinguishable from the surrounding cranial tissues. The distinct anatomical boundaries that define the otic capsule are clearly visible. These boundaries are formed by the SOs, SPs and POs, as well as the PS fissure.

#### 2.1.3 Separation of otic capsules

**FIGURE 2:**
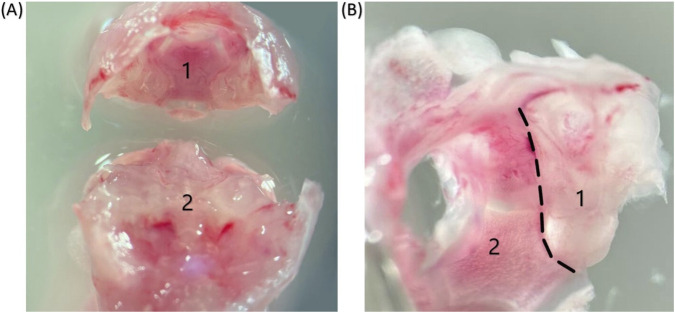
Images were obtained using a stereoscopic microscope. **(A)** The otic capsules, together with the occipital bone (1), will detach from the anterior skull portion (2) along the SOs, POs, and PS suture (magnification 16X). **(B)** Otic capsule (1) with occipital bone (2): the POs is indicated by a dashed line (magnification 25x) along which dissection is performed.

Insert Dumont #4 forceps into the cartilaginous structures of the SOs and perform gentle dissecting movements along the course of the SPs and PS suture (Figure 1B). Return the forceps to the SOs at its midline, then elevate the occipital bone posterosuperiorly. The otic capsules will detach from the anterior skull portion along both the SOs and PS suture lines (Figure 2A). Separate the otic capsule from the occipital bone along the POs (Figure 2B).

To successfully isolate the otic capsule from the temporal bone, it is very important to adjust the light source for better viewing of the surfaces of the skull base. Note that the dissection solution in the dissecting dish may become turbid and opalescent due to the release of extracellular components and intracellular structures, which significantly impairs the visualization of anatomical landmarks. To maintain clarity of anatomical landmarks, change the dissection solution as often as possible.

### 2.2 Troubleshooting

During otic capsule isolation, direct contact with the wall of the otic capsule should be minimized. Even slight pressure can damage the structures of the membranous labyrinth. Contact with dissection instruments most commonly causes cracking of the cartilaginous capsule wall. In such cases, dissection should be continued with extreme caution, applying minimal pressure to the tissues surrounding the capsule. Even if the otic capsule walls are slightly damaged, the auditory sensory epithelium and modiolus may remain intact. The lateral wall of the cochlear duct, which is reinforced by the spiral ligament, should be gently separated from the inner wall of the otic capsule.

Although the otic capsule is isolated along natural anatomical boundaries, superfluous fragments of the temporal squama, sphenoid, and occipital bones, as well as soft tissues from the cranial base and vault, may remain attached to the capsule wall. This necessitates additional removal steps, increasing the risk of mechanical damage to the otic capsule and the enclosed membranous labyrinth. The presence of cracks in the otic capsule further complicates this step.

Following complete isolation and removal of excess tissue fragments, the otic capsule displays clearly distinguishable cochlear and vestibular portions. If the vestibular part is not required, it can be carefully separated from the cochlear part. However, this must be performed with extreme caution, as the vestibular portion is anatomically continuous with the cochlear region. Following these steps, proceed with cochlear surface preparation.

### 2.3 Evaluation of isolated cochlear samples

#### 2.3.1 *Ex vivo* cochlear explant cultivation

Cochleae were isolated following the protocol detailed in this study (see Section 2.1). Integrity of the cochlear explants was assessed by immunofluorescence staining. Processing of the inner ear tissue was performed using established techniques described by Landegger et al. (2017). Explants were cultured in culture dishes (Mattek, Cat# P35G-1.5-14-C) on coverslips (Ted Pella, Cat# 26023-13) coated with poly (ethyleneimine) solution (PEI; Sigma-Aldrich, Cat# P3143). The culture medium consisted of DMEM + Glutamax medium (Gibco, Thermo Fisher Scientific, Cat# 10569010) containing 10% FBS (Gibco, Thermo Fisher Scientific, Cat# A5256701) and ampicillin sodium salt 50 μg/mL (Invitrogen, Thermo Fisher Scientific, Cat# 11593-027). Cultures were maintained for 17 h at 37 °C in a 5% CO_2_ atmosphere. Cochlear explant regions were considered morphologically intact when exhibiting preserved architecture of both inner and outer hair cell rows, with four clearly visible rows of hair cells.

#### 2.3.2 *In vitro* spiral ganglion neurons cultivation

Cultivation of the dissociated cells of modiolus was performed in a confocal culture dish coated with PEI. After the cochleae were extracted from skull and otic capsule, the outer ligament/stria vascularis and organ of Corti were dissected away from the modiolus of the cochlea that contained the spiral ganglion neurons. The samples were then incubated with a trypsin-EDTA solution (0.5%) (Gibco, Thermo Fisher Scientific, Cat# 15400054) at 37 °C for 25 min, shaken every 10 min. The enzymatic dissociation was stopped by adding 10% FBS in Ca^2+^ and Mg^2+^ -free HBSS. Spiral ganglia were mechanically dissociated by pipetting with a 200 µL pipette tip. The spiral ganglion neurons were centrifugated at 1000 rpm for 8 min, resuspended, and plated in PEI coated glass bottom dish (Mattek, Cat# P35G-1.5-14-C) at a density of 3.0 × 10^5^ cells and maintained at 37^∘^C in a humid atmosphere of 5% CO_2_. Cells were cultured for 72 h in Neurobasal Medium (Gibco, Thermo Fisher Scientific, Cat# 21103049) supplemented with B-27 (Gibco, Thermo Fisher Scientific, Cat# 17504044), GlutaMAX (Gibco, Thermo Fisher Scientific, Cat# 35050061), and antibiotic-antimycotic (100X) (Gibco, Thermo Fisher Scientific, Cat# 15240062). The mean density of β-III tubulin-positive neurons in primary culture was approximately 185 ± 24 cells per mm^2^ (mean ± SD, n = 5 dishes, three fields of view per dish).

### 2.4 Immunofluorescence staining

Cochlear explants and spiral ganglion neuron cultures were fixed in 10% formaldehyde for 30 min, permeabilized with 0.1% Triton X-100 (Sigma-Aldrich, Cat# X100) in PBS for 15 min, and blocked with 1% BSA (Sigma-Aldrich, Cat# A7906-10G) in PBS for 20 min at room temperature. The samples were incubated overnight at 4 °C with anti-β-III tubulin mouse monoclonal antibodies (dilution of 1:100) (Invitrogen, Thermo Fisher Scientific, Cat# MA1-118). Then, samples were washed with PBS and incubated for 30 min at room temperature with fluorescently labeled anti-mouse IgG (dilution of 1:200) (Sigma-Aldrich, Cat# SAB4600299). Phalloidin staining (Invitrogen, Thermo Fisher Scientific, Cat# A12379) for cochlear explants was performed by incubating explants in staining solution at room temperature for 30 min. Then, samples were washed with PBS containing 0.1% Tween-20 (Sigma-Aldrich, Cat# P7949). Cell nuclei were counterstained with DAPI (Sigma-Aldrich, Cat# D9542). Confocal imaging was performed using a Nikon Eclipse Ti2 confocal microscope equipped with a Nikon S Plan Fluor ELWD 20x/0.45 objective.

## 3 Results

**FIGURE 3:**
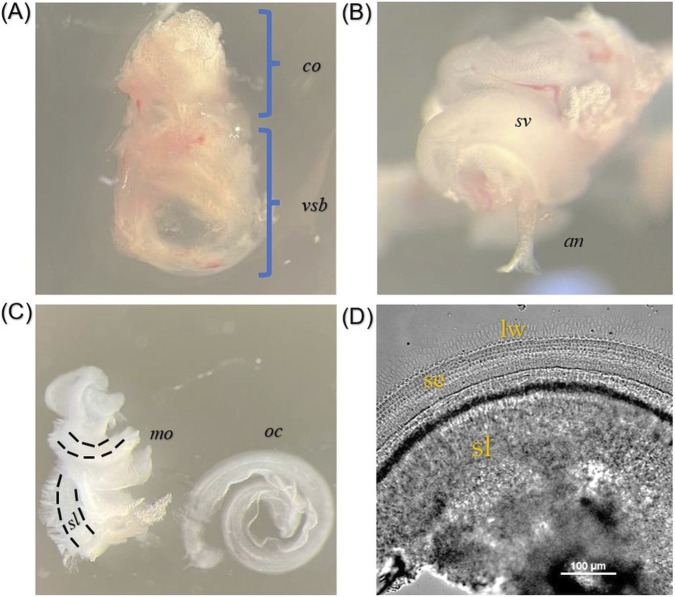
Images were obtained using a stereoscopic microscope. Intact Otic Capsule and Membranous Labyrinth Specimens. **(A)** Isolated otic capsule: co–cochlea, vsb - vestibular part of otic capsule (magnification 32X). **(B)** Membranous labyrinth: sv–stria vascularis, an–auditory nerve (magnification 50X). **(C)** modiolus and organ of Corti: mo–modiolus, oc–organ of Corti. The dashed line demarcates the spiral lamina and the enclosed spiral ganglion neurons (magnification 32X) **(D)** Cochlear explant: lw–lateral wall, se–sensory epithelium, sl–spiral lamina.

The proposed technique enables isolation of intact cochleae from within the temporal bone while preserving the integrity of otic capsule and structures of membranous labyrinth (Figure 3A). The criteria for successful cochlear isolation from the temporal bone are the absence of superfluous fragments of the temporal squama, sphenoid and occipital bones, as well as soft tissues from the cranial base and vault, which complicate subsequent processing of the inner ear tissue. Applying these criteria, the cochlea isolation success rate was 92,9% (65 successful isolations, n = 70). The cochlear isolation step, including brain extraction, takes approximately 3 min.

**FIGURE 4:**
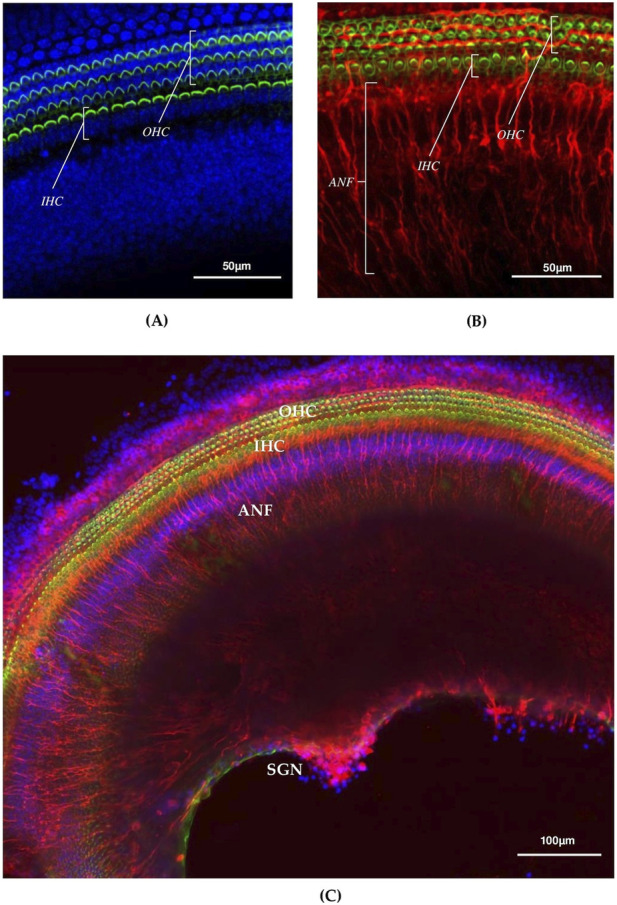
Confocal microscopy image of an intact organ of Corti explant **(A)** and cochlear explant **(B,C)** *ex vivo*. ANF–afferent nervous fibers; SGN–spiral ganglion neurons OHC–outer hair cells; IHC–inner hair cells; fluorescence staining of cochlear explant for cell markers phalloidin (HCs, green), β-III tubulin (ANF and SGN, red), DAPI (nuclei, blue). Сonfocal imaging was performed using a Nikon Eclipse Ti2 confocal microscope equipped with a Nikon S Plan Fluor ELWD 20x/0.45 objective.

The steps described in this protocol allow for minimizing the amount of surrounding tissue remaining attached to the cochlea, thereby eliminating unnecessary removal procedures. Preservation of the integrity of otic capsule and spiral configuration of membranous labyrinth increases the likelihood that all regions of the organ of Corti remain intact (Figures 3B–D). The protocol allows obtaining intact cochlear explants with preserved hair cell architecture of auditory sensory epithelium (Figures 4A–C). The preservation of organized hair cell rows and the characteristic V-shaped stereocilia bundle pattern of the cuticular plate reflects the preservation of cellular ultrastructure. It would be disrupted by significant mechanical deformation of the surrounding cochlear architecture.

**FIGURE 5:**
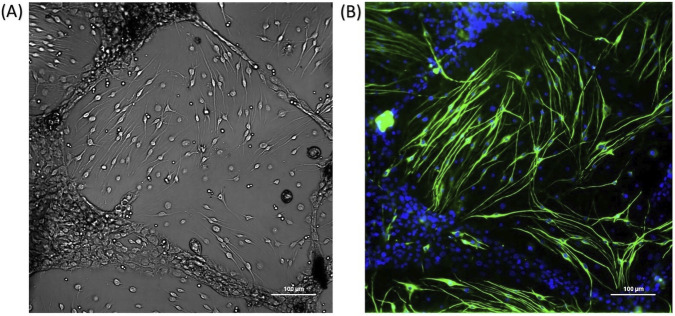
**(A)** Phase contrast image of spiral ganglion neurons in primary culture. **(B)** Immunofluorescence image of spiral ganglion neurons *in vitro*. Immunostaining for β-III tubulin (green) to visualize neuronal cells and counterstained with DAPI (blue) to identify nuclei. The spiral lamina of the modiolus, containing the spiral ganglion neuron (SGN) cell bodies, can be used to generate primary cultures, provided that the modiolus remains intact and is not mechanically damaged during the cochlear isolation procedure. Damage to the otic capsule often compromises the integrity of the enclosed soft tissues, including the delicate structures of the membranous labyrinth. Disruption of the modiolus’s spiral architecture compromises the identification of the spiral lamina, ultimately reducing the neuronal to non-neuronal cell ratio in the resulting culture.

Using this technique, isolation of a single modiolus takes approximately 7 min from decapitation. When obtaining modiolus samples for *in vitro* or *ex vivo* systems, preservation of its structural integrity is critical. Disruption of the spiral configuration of the membranous labyrinth can complicate the identification of the modiolus among surrounding tissues during processing of the inner ear tissue. The use of this approach preserves the integrity of inner ear structures, thereby maintaining the shape of the modiolus (Figure 3C). The spiral lamina, which gives the modiolus its spiral shape, contains the cell bodies of the spiral ganglion neurons. Additionally, preservation of the villous edge of the spiral lamina, which is formed by the afferent fibres of auditory neurons, facilitates the identification process of modiolus and prevents excess tissue from contaminating the experimental or culture medium (Figure 5).

**FIGURE 6:**
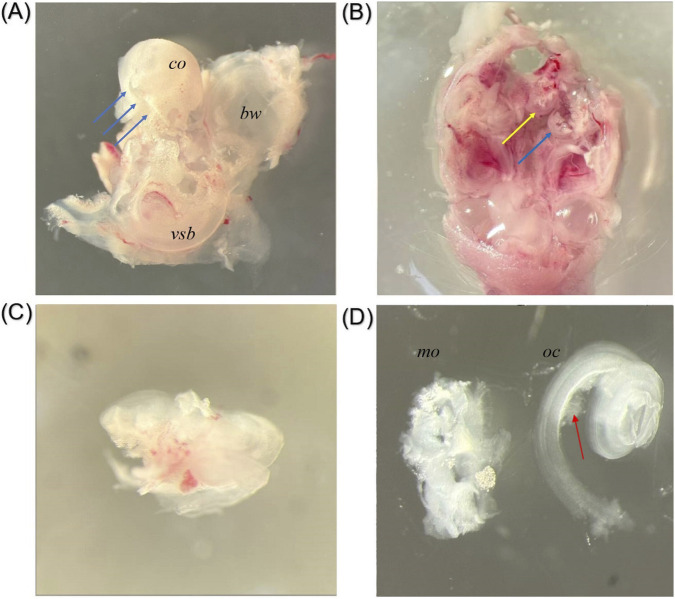
Images were obtained using a stereoscopic microscope. Damaged Otic Capsule and Membranous Labyrinth Specimens. **(A)** Cracking of the cartilaginous capsule wall. Blue arrows indicate the fracture line. Co – cochlear part, vsb–vestibular part of otic capsule, bw–bulla wall (magnification 32×). **(B)** Prolapse of the membranous labyrinth through the cracked capsule wall. The membranous labyrinth (yellow arrow) and fragments of the otic capsule (blue arrow) are indicated (magnification 16×). **(C)** Disruption of the spiral configuration of the membranous labyrinth after prolapse (magnification 50×). **(D)** Loss of spiral configuration of the modiolus; fragments of the modiolus remaining attached to the organ of Corti following extraction from a damaged specimen (red arrows). mo–modiolus, oc–organ of Corti (magnification 32×).

Following this protocol reduces the risk of the two most common types of otic capsule damage: cracking of the cartilaginous capsule wall and capsule fracture with prolapse of the membranous labyrinth (Figures 6A,B). A more severe outcome is capsule fracture with prolapse of the membranous labyrinth, resulting in loss of its spiral configuration (Figure 6C). This structural alteration complicates subsequent cochlear surface preparation. The modiolus loses its spiral shape, making it difficult to identify the basal, middle, and apical turns. Fragments of the modiolus may remain attached to the organ of Corti, which impairs explant adhesion to the culture surface (Figure 6D). Uncontrolled mechanical deformation may in turn compromise the integrity of hair cells.

## 4 Discussion

Inner ear tissues from neonatal rodents are widely used to generate experimental models *in vitro* and *ex vivo* for genetic and pharmacological research. Organotypic cultures can consist of the isolated organ of Corti or the spiral ganglion explant. Primary cell cultures can also be established from the organ of Corti or the modiolus (Wu et al., 2023; Ogier et al., 2019). Co-cultures that combine both tissue types have been described (Vincent et al., 2024). The experimental models obtained from the cochleae of neonatal animals can be studied using a wide range of methods, such as light and fluorescence microscopy, Western blot, PCR, functional imaging, patch-clamp, and many others (Hahnewald et al., 2016; Vincent et al., 2024; Haqu et al., 2015; Levano et al., 2023; Wang et al., 2021; Zhang et al., 2025). Transfection experiments on cochlear samples have been described (Parker et al., 2010).

The reproducibility of methods for establishing organotypic and primary cochlear cell cultures is highly dependent on the researcher’s skill. The time interval between animal decapitation and the incubation of organotypic cultures or isolated inner ear cells should be minimized (Parker et al., 2010). Cochlear isolation and surface preparation carry a high risk of damaging the membranous labyrinth structures. Cochlear samples can easily be damaged when transferring them to culture dishes or during other preparation steps.

The organ of Corti and spiral ganglion neurons are enclosed within the bony labyrinth of the cochlea. Therefore, accessing these structures without damaging them is a key challenge. The age of the animal is an important factor that affects both the cochlear isolation step and subsequent surface preparation. Neonatal mice have non-ossified skull bones and otic capsule. Thus, they are still soft enough for dissecting tools. Ossification of the otic capsule begins prenatally but does not complicate cochlear preparation until around 6 postnatal day (Meas et al., 2018). Therefore, existing protocols typically use animals up to 5–6 days after birth to maintain organ of Corti integrity.

Few studies have successfully cultured adult mouse cochleae. In adult mammals, the cochlea is fully ossified, so dissection is much more challenging than in neonatal tissue (Li et al., 2022). In this study, we did not evaluate cochlear isolation in postnatal ages beyond P2, as our focus was on neonatal tissue. For older ages, further optimization of the dissection approach would be required.

Although numerous studies have reported modiolus isolation in mice and rats aged P0–P5 (Buswinka et al., 2023; Levano et al., 2023; Wang et al., 2021; Zhang et al., 2025; Polikarpov et al., 2026; Peng et al., 2004), another approach is possible. Because the cochlea is still soft during this period, there is a risk of disrupting the spiral configuration of the membranous labyrinth and modiolus during isolation. According to Hahnewald et al. (2016), animals no younger than P5–P7 are preferable for spiral ganglion isolation (Hahnewald et al., 2016). The authors suggest that by this stage, the cochlear bone has undergone moderate ossification, which provides sufficient rigidity during dissection. By extension, we consider that moderate ossification of the otic capsule may also play a protective role, helping to reduce deformation during tissue preparation. This helps preserve neuronal cell bodies and dendrites within the spiral ganglion when the organ of Corti is removed. This approach is useful for researchers who want to preserve spiral ganglion cell integrity and achieve a high neuronal yield.

The critical step for preserving the auditory sensory epithelium is the removal of the stria vascularis and the spiral ligament. Removal of the organ of Corti introduces an additional risk of damage during preparation of the modiolus tissue. However, our work focuses specifically on the cochlear isolation step. Cochlear dissection for organotypic cultures must be performed as quickly as possible. Optimizing the isolation of the cochlea from the skull bones improves reproducibility and reduces time and labor. This is particularly important when preparing samples for pharmacological studies, where large numbers of cochlear explants are needed for experimental groups. For primary spiral ganglion neuron cultures, large amounts of modiolar tissue are required per experiment—in some studies, this number reaches 30–40 samples (Schwieger et al., 2015). In neonatal mice, the otic capsule is separated from the other parts of the skull by synchondroses and sutures. Therefore, this work emphasizes the technique of targeted dissection through the SOs toward the SPs and PS suture, followed by posterosuperior retraction of the occipital bone with the otic capsules. This is followed by the final stage of isolating the cochlea along the POs. The otic capsule and the enclosed membranous labyrinth are highly susceptible to mechanical deformation during dissection, particularly when separating the cochlea from the surrounding temporal bone. The proposed approach takes advantage of dissecting along the natural, non-ossified lines of the skull, which helps to reduce mechanical impact on the cochlea and consequently minimizes the risk of damage to the otic capsule during isolation.

Our results confirm the applicability of suggested cochlear isolation technique to neonatal mice. When microdissection is performed properly, the otic capsule exhibits no visible mechanical damage following cochlear isolation. The spiral structure of the membranous labyrinth and modiolus, as well as the cellular architecture of the hair cell rows, remain intact. This indicates the absence of significant mechanical deformation of the cochlea during isolation.

A limitation of this study is the absence of an assessment of how otic capsule mineralization affects the pattern of damage during isolation. Bai et al. (2023) mapped ossification foci and their dynamics from P0 to 4Mo using alizarin red staining and micro-CT (Bai et al., 2023). The authors concluded that mechanical properties of the otic capsule change across postnatal stages, which may influence damage patterns during isolation and requires further study.

## 5 Conclusion

Cochlear isolation is a technically challenging procedure that requires both practiced skill and careful execution. The proposed technique of sequential dissection through the SOs, SPs, and PS sutures, as well as the POs, utilizes targeted dissection along natural anatomical boundaries to reduce the risk of mechanical damage to the otic capsule and the structures of the membranous labyrinth. This approach also allows for the reduction of excess tissue in the dissection area, helps avoid unnecessary manipulation of the cochlea, and consequently reduces mechanical deformation caused by instrument handling. The described method has the potential to accelerate cochlear isolation, although its effectiveness largely depends on the skill and experience of the researcher. Furthermore, individual laboratories may develop their own protocol variations that offer specific advantages for different researchers. A key feature of our protocol lies in the emphasis on specific anatomical landmarks, which facilitates a clearer understanding of the cochlear isolation process. This is especially important for researchers who are developing the skills necessary to create *in vitro* or *ex vivo* cochlear models.

## Data Availability

The raw data supporting the conclusions of this article will be made available by the authors, without undue reservation.
